# Performance of a Piezoelectric Energy Harvesting System for an Energy-Autonomous Instrumented Total Hip Replacement: Experimental and Numerical Evaluation

**DOI:** 10.3390/ma14185151

**Published:** 2021-09-08

**Authors:** Hans-E. Lange, Nils Arbeiter, Rainer Bader, Daniel Kluess

**Affiliations:** 1 Department of Orthopaedics, Rostock University Medical Center, 18057 Rostock, Germany; rainer.bader@med.uni-rostock.de (R.B.); daniel.kluess@med.uni-rostock.de (D.K.); 2 Institute of General Electrical Engineering, University of Rostock, 18059 Rostock, Germany; nils.arbeiter@uni-rostock.de

**Keywords:** energy harvesting, piezoelectricity, total hip replacement, orthopaedic implant, testing, finite element analysis

## Abstract

Instrumented implants can improve the clinical outcome of total hip replacements (THRs). To overcome the drawbacks of external energy supply and batteries, energy harvesting is a promising approach to power energy-autonomous implants. Therefore, we recently presented a new piezoelectric-based energy harvesting concept for THRs. In this study, the performance of the proposed energy harvesting system was numerically and experimentally investigated. First, we numerically reproduced our previous results for the physiologically based loading situation in a simplified setup. Thereafter, this configuration was experimentally realised by the implantation of a functional model of the energy harvesting concept into an artificial bone segment. Additionally, the piezoelectric element alone was investigated to analyse the predictive power of the numerical model. We measured the generated voltage for a load profile for walking and calculated the power output. The maximum power for the directly loaded piezoelectric element and the functional model were 28.6 and 10.2 µW, respectively. Numerically, 72.7 µW was calculated. The curve progressions were qualitatively in good accordance with the numerical data. The deviations were explained by sensitivity analysis and model simplifications, e.g., material data or lower acting force levels by malalignment and differences between virtual and experimental implantation. The findings verify the feasibility of the proposed energy harvesting concept and form the basis for design optimisations with increased power output.

## 1. Introduction

Implantation of a total hip replacement (THR) is a surgical treatment for hip joint-related diseases. This final measure is taken if conservative therapies fail and cannot resolve persistent symptoms that decrease the quality of life [1]. The main indication for THR is severe hip osteoarthritis [2]; osteoarthritis in general is one of the most common disabling diseases worldwide [3,4]. Due to the increase in the world population, its aging, and a more frequent use of THR in younger patients, THR shows a proceeding rise in surgeries per year in industrialised countries, which is projected to continue [5,6,7]. With regard to the clinical outcome and economic burden, THR revision surgeries should be avoided [8,9,10,11]. However, in spite of the low revision rate, the absolute number of secondary THRs will grow due to the larger number of primary THRs and the higher demands by a longer expected service life [7,12,13]. Therefore, research is conducted for the improvement of current THRs and the reduction of existing insufficiencies. Instrumented implants with sensory or active functions can lead to increased performance of THRs. Research and development towards better THRs are promoted by monitoring implant- or patient-specific parameters, like loadings or implant loosening detection [14]. Additionally, permanent clinical evaluation may prevent failure through adequate and early action. With regard to novel therapeutic measures, active functions like stimulation for bone ingrowth (e.g., electrical stimulation [15,16,17,18,19]) are considered.

Instrumented implants raise the question of an adequate energy supply. In the beginning, percutaneous wires were used for both energy and data transmission, e.g., [20,21]. Drawbacks of wires, e.g., the potential risk of infections, led to the development of telemetric data transmission. Further disadvantages like the limited lifetime of batteries and the risk of leakage were overcome by the use of an external power supply through electromagnetic fields, e.g., [22,23]. This approach is still used in instrumented implants [24,25,26]. However, electromagnetic power transmission limits the application due to the dependence on external components and is therefore not suitable for a continuous and daily life application. A promising approach for independent and autonomous energy supply is the harvesting of energy from the environment. To the authors’ knowledge, the first researchers who proposed an energy harvesting concept for a load-bearing orthopaedic implant were Platt et al. [27]. Three piezoelectric elements were placed in the tibial tray of a total knee replacement (TKR) to transform the acting forces in the knee joint into energy by deformation of the piezoelectric elements [27]. This design was taken up by Almouahed et al., who improved it for better biomedical application with a thinner tibial tray [28,29,30]. Wilson et al. changed the position of the piezoelectric elements to the ultra-high-molecular-weight polyethylene (UHMW-PE) insert [31,32]. In different studies, Safaei et al. optimised the latter design and explored the application of the piezoelectric transducers as sensors for force measurements (total acting force and centre of pressure measurements) [33,34,35]. Although further concepts exist for TKR (e.g., [36,37,38]), Morais et al. were the first to propose an energy harvesting system for a THR [39]. The initial design consisted of a levitation-based electromagnetic power transducer in a hollowed total hip stem [39]. An extended version was proposed in 2012, where an additional rotational-based electromagnetic harvester was placed in the implant cup and in the ball component and an oscillating piezoelectric membrane was inserted into the hollowed implant ball [40].

To the best of our knowledge, no further work on energy harvesting for THRs was published until we recently proposed our new concept [41]: Here, a piezoelectric multilayer element was integrated into a total hip stem and loaded by the deformation of the implant. For this reason, a cavity was milled from the metal component. It incorporated a UHMW-PE housing that restored the former implant geometry. The housing contained the actual transducer, a piezoelectric multilayer element. During physical activity (e.g., walking) forces were acting on the THR and were partly transmitted from the hip stem through the housing on the piezoelectric multilayer element’s end faces. The generated energy is aimed to power an arbitrary instrumentation. However, the piezoelectric element itself can function as a sensor by linking its output voltage to the acting load [41]. In contrast to Morais et al., the energy harvesting is deformation-based and not movement-based [39]. We further avoided electromagnetic components to prevent any issues concerning magnetic resonance imaging (artefacts of and damage to the implant or risk for the patient by torque).

In a previous study, the endurance performance of the mechanically loaded modified metallic total hip stem was tested experimentally [42], since the fatigue properties may have been impaired by the introduced cavity. A standard endurance test based on ISO 7206–4:2010 [43] was conducted and an additional finite element analysis (FEA) of the same setup provided further information on the stress and strain distribution and local stress concentration in the cavity [42,44]. Our results in [42] showed sufficient remaining safety margin, since the test was passed by all the samples twice and the implant even endured additional loading cycles with increased force levels.

This work at hand presents the experimental results of the generated electrical output of a functional model of our proposed design to confirm its energy harvesting capabilities. A simplified and reproducible testing setup was developed. The completely assembled functional model was implanted into a biomechanical artificial bone segment embedded in a specimen holder and loaded by a uniaxial testing machine with a force profile based on physiological loading patterns from in-vivo measurements [45]. The alignment of the femoral bone segment in the testing machine and the force scaling were defined numerically by a preceding iterative finite element (FE) study to reproduce the previously numerically investigated physiologically based loading situation [41].

## 2. Materials and Methods

First, the FE model is described, which was used to define the alignment and forces for the experimental setup and numerically calculate the resulting power. Thereafter, our experimental setup and evaluation procedure are presented.

### 2.1. Finite Elemente Analysis

The general FE model setup can be found directly below. The different analyses are briefly described at the end of this section, in particular the iterative study and the sensitivity analysis.

#### 2.1.1. Geometry

**Figure 1:**
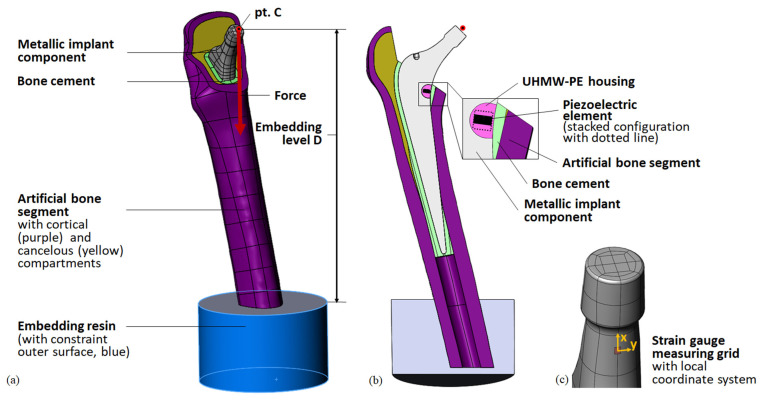
Geometry of the model for the simplified testing setup. (**a**) General view with loading and boundary conditions for the FEA; (**b**) cross-sectional view with detail of the energy harvesting system; (**c**) detail of the metallic implant component (neck) with surface patch representing the stain gauge’s measuring grid (red) and local coordinate system for evaluation of the strain.

The implant geometry was derived from a previously 3D-scanned and reconstructed arbitrary cemented total hip stem (Exeter V40, size 37.5 mm N°3, Stryker, Howmedica Osteonics Corp, Mahwah, NJ, USA) [41]. All geometrical modelling was performed using SolidWorks 2018 (Dassault Systèmes, Vélizy-Villacoublay, France). The metallic prosthetic head was not modelled but was represented by the ball’s centre of rotation point *C.* Its position reflects the maximum allowed offset of 8 mm, prescribed by the manufacturer, resulting in the maximum lever arm of the acting hip contact force. From the medial part of the total hip stem, a cavity was excised. It contained the UHWM-PE housing in which the ring-shaped lead zirconate titanate (PZT) off-the-shelf piezoelectric multilayer element was placed (Figure 1b). As in our previous work, we studied a single piezoelectric element (PICMA^®^ actuator, provided by PI Ceramic GmbH, Lederhose, Germany; outer diameter 5 mm, inner diameter 2.5 mm, height 2.5 mm, capacity 110 nF) and a stacked configuration of two elements (outer diameter 5 mm, inner diameter 2.5 mm, height 5 mm, capacity 220 nF). According to the manufacturer, a single piezoelectric element consists of 43 active layers (ca. 0.05 mm layer thickness) and a passive top and bottom layer (ca. 0.175 mm layer thickness). For the stacked configuration, two single elements were piled. The design and configurations of the integrated energy harvesting system were identical to our initial work [41] and are shown in Figure 1. To compare the numerically calculated implant loading with the experimental results, a strain gauge was modelled. A surface patch was generated on the implant’s lower neck, representing the measure grid of the strain gauge (see Figure 1c).

The total hip stem was virtually implanted into the CAD geometry of an artificial bone segment (4th generation, large left, composite bone, solid foam core, Sawbone Europe AB, Malmö, Sweden), generated by Wieding et al. [46]. For the full femoral bone, landmarks (head centre, femoral notch, epicondyles, neck axis, and femur axis) and a femur-based coordinate system according to Bergmann et al. [45] were defined. The implant’s virtual centre of rotation point *C* was superimposed with the head centre of the bone. The distal tip of the total hip stem was placed on the femoral axis and the neck axes of both components were defined to lie in the same plane. The femoral head resection was comparable to the previous simulation of a human femur, maintaining the greater trochanter [41]. The bone cement layer was initially defined as a 3 mm-thick layer around the total hip stem. For the generation of the bone cavity, it was subtracted from the cancellous bone compartment. Since the reamers for bone preparation cannot remove material from the artificial cortical bone, the former homogeneous cement mantle was reduced by the intersecting volumes. A small volume of the cortical bone had to be subtracted due to overlaps with the total hip stem geometry. Therefore, in contrast to ideal cementation, the bone cement mantle was not surrounding the whole implant, but good accordance with the realised implantation was prioritised (see Section 2.2.1 and Section 3.2). Finally, the bone cement mantle in the lower part was augmented to fill up the persisting void in the medullary cavity around the implant. See Figure 1b for a cross-sectional view of the virtual implantation. The overall virtual implantation was consistent with the implantation in our preceding study [41].

The femoral bone was virtually embedded into a specimen holder. As a simplification, only the embedding resin was modelled. The top surface of the cylindrical component was placed at distance D (embedding level, final value after iterative study D = 199 mm) from point *C* and its midpoint was superposed with the extended total hip stem axis to centre the femoral bone segment in the specimen holder. The cylinder axis of the embedding resin was defined to be parallel to the acting force direction. Thus, the femoral bone was aligned with the specimen holder, simulating the placement in a uniaxial testing machine. Finally, the femoral bone was cut 5 mm above the lower surface of the embedding resin. The cavity for the remaining bone segment in the embedding resin was created by a Boolean operation. See Figure 1 for the full model.

#### 2.1.2. Loads and Boundary Conditions

The FEA was carried out in ANSYS Workbench V19.2 (Ansys Inc, Canonsburg, PA, USA). The model was loaded in the remote point *C* in the femur-based coordinate system with scaled force components from Bergman et al. [45] for the instance of maximum total hip contact force during the walking gait cycle for a patient of an average weight of 75 kg. The final force values are shown in Table 1 (see also Table 6 for the data used from Bergmann et al.). The force was distributed from point *C* to the outer surface of the taper and was acting in the direction of the cylinder axis of the embedding resin (see Figure 1a).

The outer surface of the embedding resin was fully constrained, except its top surface, simulating a fixed specimen holder.

The inner surfaces of the femoral bone and the outer surface of the bone cement mantle were connected by a bonded contact. Thus, no relative movement was possible. All other contacts were defined to be frictional, in particular between the metallic component of the implant and the bone cement (µ = 0.35 [47]) and the cortical bone (assumed µ = 0.35) and between the metallic implant component and the UHMW-PE housing (µ = 0.15 [48]). For the remaining contacts, a friction coefficient of µ = 0.3 was assumed (from the UHMW-PE housing towards the bone cement and the piezoelectric element and finally between the cortical bone and the embedding resin). The cortical and cancellous bone compartments and all piezoelectric element layers were treated as single components with a continuous FE mesh without the need for contact definitions.

For the active layers of the piezoelectric element, an alternating polarisation behaviour in the direction of or reverse to the cylinder axis of the element was defined. The electric interconnection was reproduced by simulating two different electrodes. The potential degree of freedom of consecutive layer surfaces was coupled to either a common floating potential or a ground potential. This reproduced the general setup of a piezoelectric multilayer element.

#### 2.1.3. Meshing

For the piezoelectric element layers, a structured hexahedral mesh was generated to account for a minimum number of elements over their thin height (quadratic SOLID226 elements for active layers, quadratic SOLID186 elements for passive layers). All other more complex solid geometries were meshed with quadratic tetrahedral elements (SOLID187). The strain gauge was represented by a single linear shell element (SHELL181), with corner nodes defined by the surface patch described before.

The final mesh density was based on a mesh independence study. The convergence of the global stiffness characteristics was verified by the displacement of point *C* $d_{C,\mathrm{total}}$ . A combined approach of global and additional local mesh refinement was chosen to enable the convergence of all the reported output parameters. The final mesh was re-coarsened for the additional studies to reduce the numerical cost. The maximum deviation from the densest mesh was <2% for all the output parameters.

#### 2.1.4. Material Properties

For all components, linear–elastic and isotropic material behaviour were assumed. The material properties are listed in Table 2.

The piezoelectric properties of the PZT material were provided by the manufacturer (see Table 3) and were used to define the active layers in ANSYS Workbench V19.2 and for the post-processing (see below) [51]. They are in accordance with data from the literature [55].

#### 2.1.5. Analysis Settings and Hardware

The simulations were performed on a high performance computing Linux cluster (each node with two Intel^®^ Xeon^®^ Gold 6248 CPU processors (2.50 GHz) and 192 GB RAM) using a sparse direct solver. Regarding the convergence of the solution, the total force was applied incrementally starting with a step size of 10%. Due to the frictional contact definitions, non-linear behaviour was assumed.

#### 2.1.6. Post-Processing and Output Parameters

To compare the new setup to our previous physiologically based reference simulations [41], we considered the following output parameters:

- maximum von Mises stress in the implant cavity σ_imp_,
- maximum von Mises stress in the piezoelectric element’s midplane cross-section σ_piez_ (i.e., for the stacked configuration, the higher stress value of the two midplanes of the two single elements),
- contact force F_33_ acting on the piezoelectric element’s end faces in the direction of its cylinder axis,
- generated open-circuit voltage V_OC,_ and
- total displacement of point *C* $d_{C,\mathrm{total}}$ .

Regarding validation and the experiment, we also evaluated the displacement of point *C* in vertical direction of the uniaxial testing machine (direction of the acting force) $d_{C,v}$ and the strain $\varepsilon_{\mathrm{SG}}$ in the simulated strain gauge in the measuring direction for a stepwise applied maximum force F_total_ (10 force increments).

For different load resistances R, we calculated the generated voltage v(t) and the average power P for 10 gait cycles based on the work of Wilson [32] and Safaei [56,57] using MATLAB 8.4 R2014b (MathWorks Inc., Natick, MA, USA). The load profile from Bergmann et al. for walking [45] was scaled to the simulated force F_33_, acting on the piezo element’s end faces at the instance of maximum total hip contact force. We introduced a force rise from zero for the first cycle to correspond to the experiment (see Figure 8 for the exemplary scaled load profile for the stacked configuration). The approximated load profile for F_33_(t) was input to solve the differential Equation (1) for the piezoelectric element at hand with the capacity C_piez_ and the number of layers n_layer,_ connected to a single load resistance R. For the derivation of the equation, see the work of Safaei [56,57].

(1) $$C_{\mathrm{piez}}\frac{\mathrm{dv(t)}}{\mathrm{dt}}+\frac{v\left(t\right)}{R}=n_{\mathrm{layer}}\;d_{33}\;{\dot{F}}_{33}\left(t\right)$$

The capacity C_piez_ was provided by the manufacturer (single element 110 nF; stacked element 220 nF with ±20% tolerance [58]). It can also be calculated by

(2) $$C_{\mathrm{piez}}=\frac{n_{\mathrm{layer}}\varepsilon_{33}^{T}A}{\frac{h}{n_{\mathrm{layer}}}}$$

where A defines the base area of the piezoelectric element and h the total height of the piezoelectric active volume [56]. This approach was chosen by Safaei et al., resulting in notably higher capacities C_piez_ of 168.9 nF for a single element and 337.7 nF for the stacked element (see Table 4).

Based on the voltage v(t) for n = 10 gait cycles, each of duration T, the power was calculated using

(3) $$P=\frac{1}{T*n}\int_{0}^{T*n}\frac{v²\left(t\right)}{R}\mathrm{dt}$$

#### 2.1.7. FEA Studies

##### Iterative Study for the Reproduction of Previous Results in the New Setup

The iterative study was performed for the single piezoelectric multilayer element, starting with the original force components for a patient of an average weight of 75 kg from Bergmann et al. [45] (see Table 6) and a first guess embedding level D. We successively scaled the force components and the embedding level to minimize the deviation of the considered output parameters (σ_imp_, σ_piez_, F_33,_ V_OC_, and $d_{C,\mathrm{total}}$ ) from our previous work [41]. The final setup settings were also used to simulate the stacked piezoelectric element configuration. We aimed for differences ≤3%.

##### Sensitivity Analysis

We performed a sensitivity analysis to investigate the influence of input parameters on the output parameters relevant to the experimental testing ( $d_{C,v}$ , F_33_ (influencing the generated voltage v(t)), P, and $\varepsilon_{\mathrm{SG}}$ ). Regarding the mechanical safety of the system, we also evaluated the stresses σ_imp_ and σ_piez_. As a reference model, we used the configuration with a stacked piezoelectric element, which was also used in the experimental testing because of the higher power output. Particularly, we considered

- geometrical changes
  ○ resection level of the bone (distance to energy harvesting system); –1, +1, +2 mm
  ○ thickness of the bone cement layer; ±1 mm
  ○ embedding level D; ±3 mm
  ○ alignment of the femoral bone segment; ±5° rotation in the implants’ medio-lateral plane and antero-posterior plane
- scaled material properties (±10% Young’s moduli for artificial bone, bone cement, housing and metallic total hip stem).

### 2.2. Experimental Testing

The configuration of a stacked piezoelectric multilayer element (height of 5 mm) was chosen for the experimental evaluation, based on the results from the FEA. Compared to the single element, it provided higher generated voltage v(t) and more power output for the same loading settings. The sample preparation is described below, followed by the different testing regimes.

#### 2.2.1. Sample Preparation

**Figure 2:**
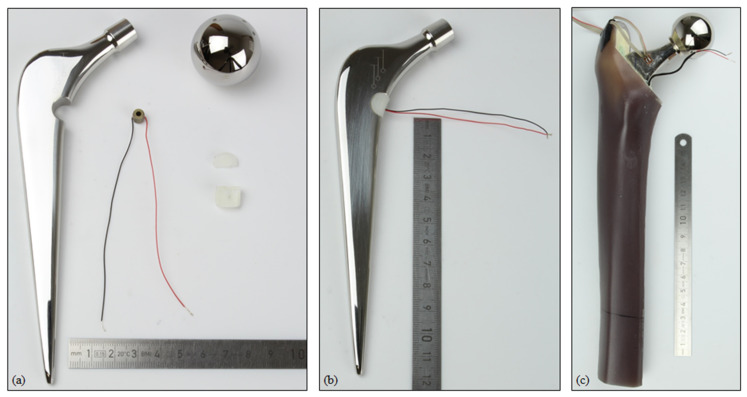
(**a**) Single parts of the modified total hip stem with the energy harvesting system (metallic implant component, prosthetic head, piezoelectric element, UHMW-PE housing in two pieces); (**b**) fully assembled system; (**c**) implanted system within the artificial bone segment, with the wires from the strain gauge and piezoelectric element visible.

The modified total hip stem geometry was created by milling from an original implant by an external manufacturer. Similarly, the UHMW-PE housing was processed from bulk material. To insert the piezoelectric element, the housing geometry was split into two pluggable pieces. All single parts and the assembly are shown in Figure 2.

A linear strain gauge (DMS 1.5/120 LY15, Hottinger Baldwin Messtechnik GmbH, Darmstadt, Germany) was applied to the lower neck of the hip stem. The position was in accordance with the simulation and the positioning was realised through an additive manufactured template based on the CAD geometry. To account for temperature effects during the measurement, an additional strain gauge was applied to a second and equal implant, which was unloaded. These data served for temperature compensation.

The best possible reproduction of the virtual implantation was attempted. Therefore, the geometry to resect was transferred to the artificial bone from the CAD model. The femoral head and the distal part were sawn off. The implantation was performed according to the manufacturer’s instructions. With a broach from the original surgery equipment, the bone cavity was excised. To prevent bone cement and embedding resin from pouring into the empty medullary cavity, it was sealed with a small amount of modelling clay. Next, the bone cement (PALACOS^®^, Heraeus Medical GmbH, Wehrheim, Germany) was prepared according to the manual. It was introduced into the bone cavity and the assembled total hip stem with the energy harvesting system was implanted by an experienced orthopaedic surgeon. To achieve an optimal position of the distal tip, the winged centraliser was used. The wires from the piezoelectric element to the resistors and measuring equipment were guided through the bone cement. To prevent cable breakage, they were tubed by a soft hose. The implant was kept in position until the polymerisation was finished. 

The prosthetic head (diameter: 32 mm, +8 mm offset) was mounted on the total hip stem and the full system was oriented towards the specimen holder with the help of an alignment fixture. The alignment in accordance with the CAD model was based on landmarks of the total hip stem (symmetry plane and axis defined by the introducer hole and distal tip). Thereafter, the specimen holder was filled with embedding resin (RenCast^®^ FC 52/53 Isocyanate/FC 53 Polyol, filler DT 082; Huntsman Advanced Materials GmbH, Basel, Switzerland) to the specified embedding level.

**Figure 3:**
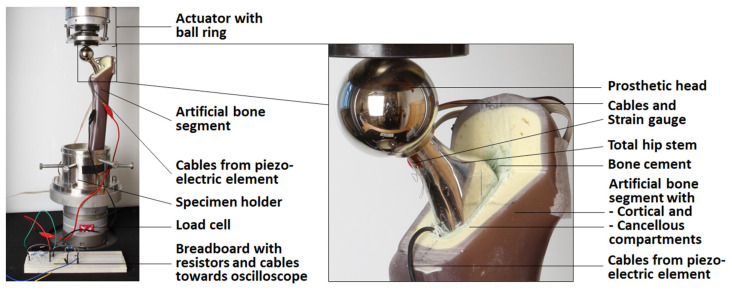
Test setup of the full system with detailed view of the implant (only the actuator of the testing machine is shown; other equipment is described in the text).

The whole sample was positioned in an electro-dynamic uniaxial testing machine (ElectroPuls E3000, Instron, Norwood, MA, USA with a 5 kN load cell). To prevent the transmission of lateral forces, a ball ring was integrated between the actuator and the prosthetic head. The overall setup of the implanted total hip stem in the testing machine is shown in Figure 3.

#### 2.2.2. Quasi-Static Testing for Model Validation

To compare the mechanical behaviour of our experimental setup with the FEA, we studied the vertical displacement of the prosthetic head $d_{C,v}$ (equals actuator displacement) and the measured strain gauge strain $\varepsilon_{\mathrm{SG}}$ for quasi-static testing. The specimen was preloaded with 5 N, and the maximum total force of 1892.99 N was applied stepwise in 10 increments. Each load level was held for five seconds. For this period, the measured strain $\varepsilon_{\mathrm{SG}}$ and the displacement of point *C* in vertical direction $d_{C,v}$ were averaged. The experiment was repeated three times.

#### 2.2.3. Mechanical Testing

**Figure 4:**
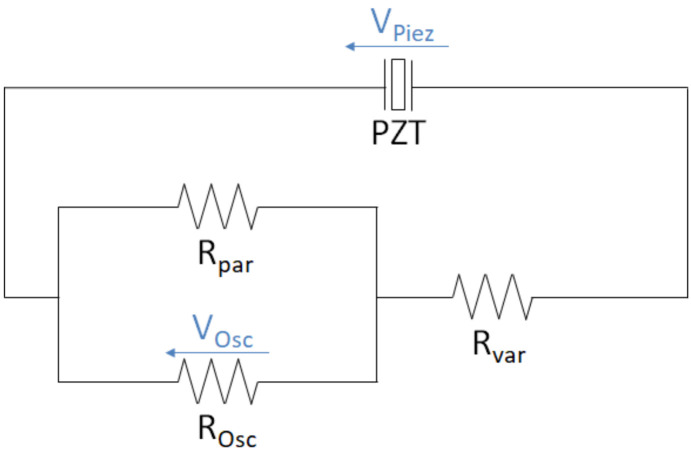
Measuring circuit of the piezoelectric element (PZT) for different load resistances with a voltage divider and an oscilloscope.

For the mechanical testing, the system was again preloaded with 5 N. Next, the force profile of Bergmann et al. for walking [45] scaled to the maximum total force of 1892.99 N was applied for n = 10 cycles with a period of T = 1.1229 s (see Figure 7 for the load profile). The voltage $V_{Osc}$ (t) for different total load resistances (R = 0.1 to 3.0 MΩ) was measured with an oscilloscope (R&S^®^RTB2004, Rohde & Schwarz GmbH & Co. KG, Munich, Germany) across a fixed resistor R_par_ of a voltage divider with the sampling period t_s_ = 0.137 ms. The fixed resistor had a relatively low resistance (R_par_ = 10101 Ω) compared to the internal resistance of the oscilloscope (R_Osc_ = 1 MΩ), together resulting in a 10 kΩ equivalent resistance. The measuring circuit was set up on a breadboard and is shown in Figure 4.

The post-processing of the data was done in MATLAB 8.4 R2014b. The measured signal was smoothed to reduce signal noise and the total generated voltage $V_{Piez}\left(t\right)$ was calculated by

(4) $$V_{Piez}\left(t\right)=V_{Osc}\left(t\right)*\left(1+R_{var}\frac{R_{Osc}+R_{par}}{R_{Osc}*R_{par}}\right)$$

For each total load resistance R, the power output was calculated as:

(5) $$P=\frac{1}{T*n}\sum^{}\frac{V_{Piez}{\;}^{2}\left(t\right)}{R}t_{s}$$

#### 2.2.4. Testing of the Piezoelectric Element 

Additional testing of the piezoelectric element alone was performed to evaluate the predictive power of the numerical model used and to compare with the experiment of the implanted configuration. Therefore, the stacked piezoelectric multilayer element was directly loaded by the testing machine with a force profile scaled to the maximum contact force F_33_ of 194.93 N derived from the FEA. In other respects, all steps were performed according to Section 2.2.3.

## 3. Results

The results of the FEA include the deformation and loading analysis for the final setup of the iterative study and the data of the sensitivity analysis. Next, the experimental results are presented along with data from the FEA for validation.

### 3.1. Results of Finite Element Analysis: Deformation, Loading, and Sensitivity

**Figure 5:**
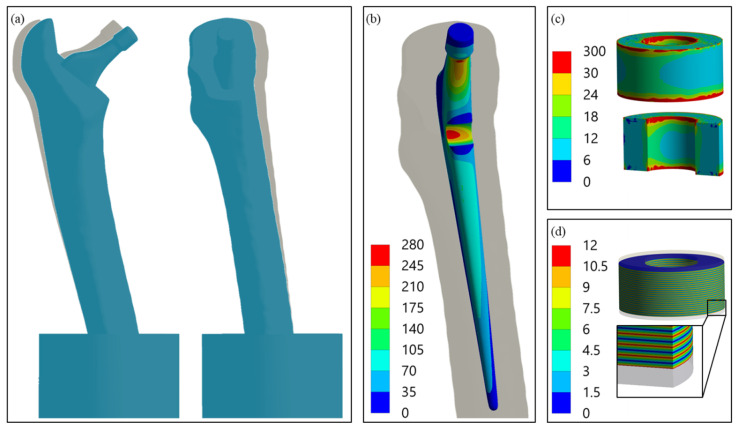
(**a**) Deformation of the whole system with non-deformed geometry in the background, 10-fold scaling for clarity, postero-anterior view (left) and latero-medial view (right); (**b**) Von Mises stress distribution (MPa) in the modified implant geometry with maximum at the introduced cavity base; (**c**) Von Mises stress distribution (MPa) in the piezoelectric element with a cross-sectional view, areas of excessive stress due to contact boundary conditions are shown in red; (**d**) electric potential distribution (V) in the active piezoelectric multilayer component with a cross-sectional view of the detail.

For a general understanding, Figure 5 shows the results of the FEA exemplarily for the single element configuration (final model after the iterative study). The deformation behaviour can be characterised by the displacement of point *C*, representing the ball’s centre of rotation. It moved distally towards the embedding resin ( $d_{C,v}$ = 0.48 mm) and forced the proximal artificial bone segment to bend in the lateral and posterior directions (Figure 5a). As for our previous work, the stress distribution showed a concentration at the cavity base, which was also the global maximum for the metallic implant component (σ_piez_ = 273.5 MPa). The piezoelectric element was relatively homogenously compressed, apart from the top and bottom end face, where influence of the contact boundary conditions resulted in a local stress rise (please note the scaling of the legend for Figure 5c). This effect faded after a few elements. For the undisturbed midplane, the stress σ_piez_ amounted to 16.6 MPa. The electrical interconnection and polarisation of the piezoelectric element’s layers resulted in the alternating distribution of the electrical potential. The simulated open-circuit voltage V_OC_ was 11.6 V.

The output parameters resulting from the iterative study are shown in Table 5. More than 150 different design points were investigated to successively minimise the differences to the output values of our previous study [41]. The finally chosen loading situation was also used to simulate the stacked configuration. Apart from the stress in the piezoelectric element σ_piez_, the aimed difference of ≤ 3% was achieved. For the stacked configuration, the results were even better, with relative differences of ≤ 1% . The stress in the piezoelectric element’s representative undisturbed midplane σ_piez_ was higher for both configurations compared to the previous work (17.7% for the single element and 7.1% for the stacked configuration) and the aimed value of ≤ 3% was not achieved. No further decrease could be realised without impairing the results of the open-circuit voltage V_OC_ or the contact forces F_33_. However, the absolute values for the deviation were small (<3 MPa).

The scaling of the force components for the final configuration is shown in Table 6. To reproduce the loading situation from the previous work, the absolute values of the x- and y-component needed to be strongly reduced. In contrast, the z-component was slightly increased. However, the difference between the total force values was only 1.8%.

The maximum power output calculated for the single element amounted to 18.4 µW for a resistance of 1.35 MΩ and 72.7 µW for a resistance of 0.68 MΩ for the stacked configuration. The calculated voltage slope for the two gait cycles and the power for different total load resistance are shown together with the corresponding experimental data further below for the stacked configuration (Figures 8–10).

**Figure A1:**
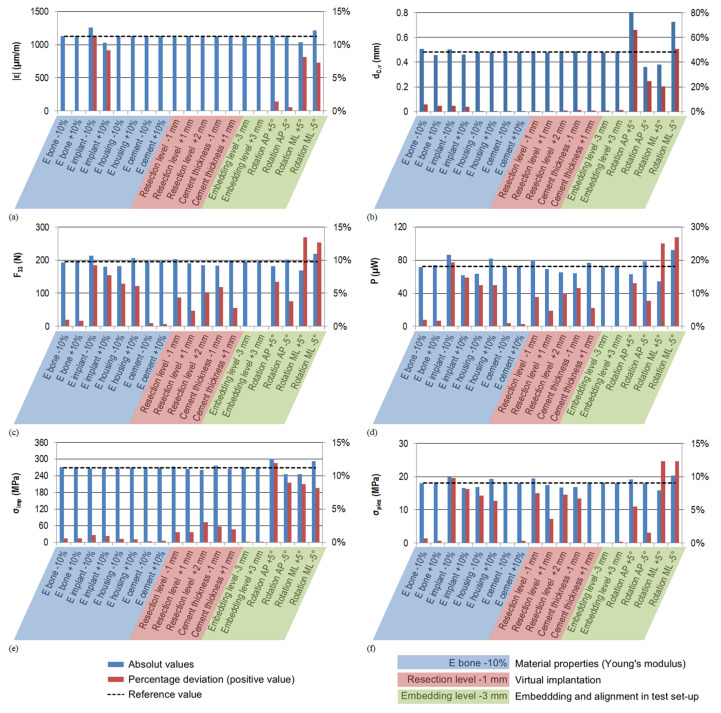
Results of the sensitivity analysis (absolute values in blue and percent deviation in red). The dashed line represents the original value of the reference model. For convenience, the absolute values of the percentages are shown. (**a**) Simulated strain for the strain gauge $\varepsilon_{\mathrm{SG}}\;$ (µm/m); (**b**) displacement of point C in vertical direction of the uniaxial testing machine (direction of the acting force) $d_{C,v}$ (mm); (**c**) contact force F_33_ acting on the piezoelectric element’s end faces in the direction of its cylinder axis (N); (**d**) generated power calculated from F_33_ (µW); (**e**) maximum von Mises stress in the implant cavity σ_imp_ (MPa); and (**f**) maximum von Mises stress in the piezoelectric element mid plane cross-section σ_piez_ (MPa). (Abbreviations: E—Young’s modulus; AP—antero-posterior; ML—medio-lateral).

The results of the sensitivity analysis are described below, revealing the relevant input parameters influencing the output. The full result data are shown in Figure A1, Appendix A.1. Regarding the experimental validation, the strain gauge strain $\varepsilon_{\mathrm{SG}}$ , the displacement of point *C* in vertical direction $d_{C,v}$ and the power output were evaluated. In the sensitivity analysis, the simulated strain gauge strain $\varepsilon_{\mathrm{SG}}$ was unaffected by nearly all the parameters but the implant material and the alignment. The ±10% change in the Young’s modulus of the implant led to an equal change (–9 to 11%) of the strain $\varepsilon_{\mathrm{SG}}$ . The higher Young’s modulus resulted in a lower strain and vice versa. The influence of rotation in the antero-posterior plane was lower (–0.6 to 1.4%) compared to the change of –8 to 7% for rotation in the medio-lateral plane.

The displacement of point *C* in vertical direction $d_{C,v}$ was the only parameter slightly sensitive to the change in the embedding level (±1%). In a comparable range, the influence of the cement mantle thickness and resection level could be found. The variation in Young’s moduli of the artificial bone and implant of ±10% resulted in a change of ±4 to ±5% of $d_{C,v}$ . With –25 to 66% for the antero-posterior rotation and –20 to 51% for the medio-lateral rotation, the alignment had the most pronounced sensitivity towards $d_{C,v}$ .

The power output was calculated on the basis of the contact force F_33_ acting on the piezoelectric end faces, which is therefore presented first. The results of the sensitivity analysis show that the contact force F_33_ and the open-circuit voltage V_OC_ were identically influenced; therefore, only the contact force F_33_ is presented and shown in Figure A1, Appendix A.1. The 10% change in the stiffness of the implant or UHWM-PE housing changed the contact force in a range of up to 10%, respectively up to 6%. Lower stiffness of the implant and higher stiffness of the housing increased the contact force F_33_. The resection level more distally (i.e., the energy harvesting position closer to the cut) increased the loading (by around 4%). A more proximal resection level decreased the force level by up to –5%. With –6%, a thinner cement mantle showed a relevant sensitivity, and a higher thickness resulted in a rise of +2.5%. The contact force F_33_ was smaller (–7%) for a higher angle in the antero-posterior plane and vice versa. The highest sensitivity for this parameter was for the rotation in the medio-lateral plane with ±13%. The sensitivity of the power output for each configuration was twice as high compared to the sensitivity to the force F_33_. The change in the artificial bone’s material data had an influence of around ±2%, for the UHWM-PE housing it was ±12%, and for the implant up to 19%. Different resection levels changed the power output by up to –10% (more proximal) or 9% (more distal). A thinner cement mantle reduced the power by 12% and a thicker geometry increased it by 6%. The influence of alignment was again most pronounced, with –13 to 8% for rotation in the antero-posterior plane and –25 to 27% in the medio-lateral plane.

For the stress concentration in the cavity base σ_imp_, changes in the material’s Young’s moduli by ±10% resulted only in changes of around ±1% or less. A slightly higher sensitivity was shown by changing the resection level: With a +2 mm more proximal cut, the stress σ_imp_ decreased by 3%. In contrast, a rise of 1.5% resulted from resecting more distally and nearly directly above the cavity. The change in the cement mantle geometry by 1 mm resulted in a stress rise of 2% for a thicker cement mantle and a decrease for a thinner cement mantle. With 8–12% of relative change, the rotation in the medio-lateral plane and antero-posterior plane had the highest influence on the loading in the cavity base, with a maximum rise by 32.0 MPa to a total value of 301.6 MPa.

Regarding the changed material properties, the stress in the piezoelectric element σ_piez_ was influenced by the Young’s modulus of the implant (up to 10%) and the UHMW-PE housing (up to 7%). A decreased stiffness of the implant and an increased stiffness of the UHMW-PE raised the stress. A resection level more distally increased the stress σ_piez_ (by 8%), whereas the stress deceased for the thinner cement mantle and the more proximal resection levels by 7%. The highest sensitivity was shown for the rotation in the medio-lateral plane (±12%), with a maximum value of 20.4 MPa. In the antero-posterior plane, it amounted to up to 6% only. In the antero-posterior plane, the effect of rotation on the stress σ_piez_ was inverse to the change in the contact force F_33_. Although a higher angle increased the stress σ_piez_, it reduced the acting contact force F_33_ and vice versa.

### 3.2. Experimental Results and Validation

**Figure 6:**
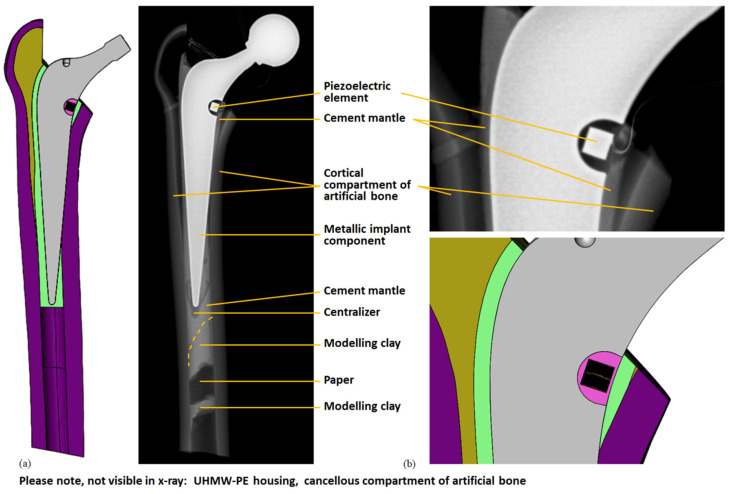
Comparison of virtual and realised implantation. Cross-sectional view of the CAD model (cf. Figure 1) and X-ray image: (**a**) energy harvesting system with artificial bone; (**b**) detail of the energy harvesting system. The cables from the piezoelectric element and strain gauge are also visible. The modelling clay and paper were used to prevent the cement from pouring too far into the medullary cavity and the centraliser assured a central position of the distal implant tip; these parts were not contained in the CAD model.

The implantation result was analysed under X-ray (X-ray unit: Gierth HF80ML Ultra Light, GIERTH X-Ray international GmbH, Riesa, Germany; digital detector system: Leonardo DR 1210, Oehm und Rehbein GmbH, Rostock, Germany) and compared to the CAD model. The cross-sectional view showed good accordance (see Figure 6). In particular, the position of the energy harvesting system to the resection level matched well under visual inspection. The overall cement mantle geometry was consistent with the CAD model; however, in the proximal total hip stem region, it was slightly thinner for the experimentally realised implantation. A portion of the lateral implant surface was uncovered by the cement mantle and was in direct contact with the cortical geometry for both the virtual and the realised implantation.

**Figure 7:**
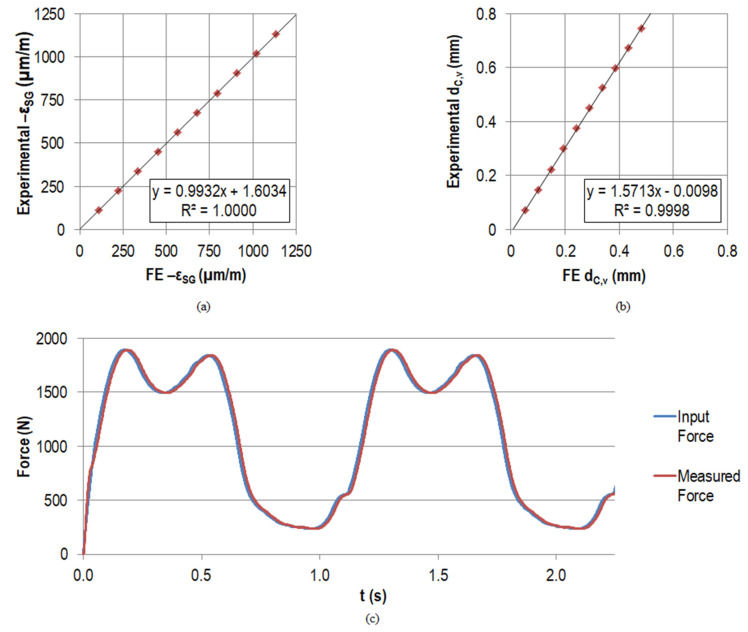
Linear regression results between FE data and experimental measurements of the strain gauge strain $\varepsilon_{\mathrm{SG}}$ (µm/m) in (**a**) and of the displacement of point C in vertical direction $d_{C,v}$ (mm) in (**b**) when applying the maximal force of the gait cycle step-wise in 10 increments. All the measured and simulated strains were of compressive nature (negative strain value); for convenience, the axes show $-\varepsilon_{\mathrm{SG}}$ . In (**c**), the force input and the measured force by the load cell are displayed for the first two load cycles (N).

The averaged measured maximum strain $\varepsilon_{\mathrm{SG}}$ at the maximum force level was –1134.0 µm/m with a standard deviation of 0.3 µm/m (equals 0.03%) for the three repetitions. This compressive strain increased by approximately –113.5 µm/m per force increment, and the maximum standard deviation of 0.9 µm/m was found at the penultimate load level. The measured strain levels for the holding time of 5 s for the individual force levels were stable with a maximum standard deviation of 0.1 µm/m. The percent deviation compared to the simulated strain was <0.68% for each force level (apart from the initial situation with 5 N preload). At the maximum force level, the deviation amounted to 4.3 µm/m (equals 0.38%). The good accordance was also reflected by the linear regression between the experimental and measured data as depicted in Figure 7a, with a slope of 0.9932 and a coefficient of determination R² of 1.0000.

The vertical displacement of the actuator corresponded to the displacement of point *C* in vertical direction $d_{C,v}$ . The average measured maximum value at the maximum force level amounted to 0.74 mm with a standard deviation of 0.0019 mm (equals 0.26%), which was also the maximum standard deviation for all force levels after the preload. The measured displacements $d_{C,v}$ for the holding time of 5 s for the individual force levels had no large fluctuations with a maximum standard deviation of 0.0005 mm; it was always below 0.3% of the mean value. Regarding the simulated displacements $d_{C,v}$ , the experimental values for each force level were approximately 1.5 times higher (see Figure 7b). Therefore, the linear regression line had a slope of 1.5713. The coefficient of determination (R² = 0.9998) was comparably high to the coefficient of determination for the strain $\varepsilon_{\mathrm{SG}}$ . The absolute maximum deviation occurred for the highest force level with 0.2598 mm (53.76% compared to the simulated displacement $d_{C,v}$ ).

The loads measured by the load cell for the different force levels were in accordance with the input data. Deviations were <0.003% for the averaged forces at all levels for the three repetitions. The standard deviation at the individual force levels for the holding time of 5 s per level was always <0.15 N. The good accordance of the force input and the measured force also for the dynamic testing is reflected by the graph in Figure 7c. The percent deviation between the absolute maximum and minimum for an arbitrary cycle was <0.6% and <0.3%, respectively.

**Figure 8:**
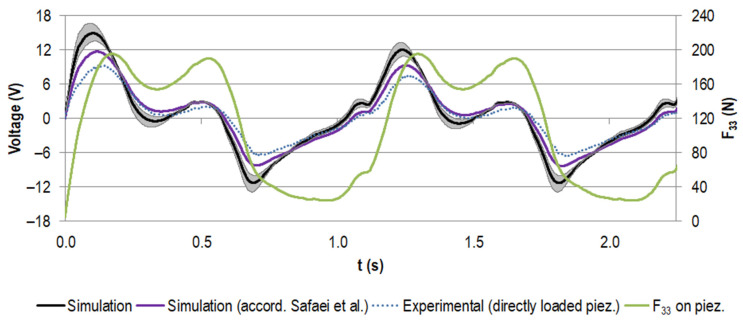
Generated voltage (V) for the first two gait cycles (time t in s) for the stacked configuration at a load resistance R of 0.5 MΩ, assuming the capacity provided by the manufacturer (in black, with tolerance of ±20% in grey) and the capacity calculated according to Safaei et al. [56,57] (in purple). Experimental data for the directly loaded piezoelectric element are shown in blue (dotted line). The scaled load profile F_33_ (N) as the basis for the simulation and as input for the uniaxial testing machine is displayed in green.

Figure 8 displays the simulated voltage for the stacked piezoelectric multilayer element, assuming the nominal capacity from the manufacturer with the given tolerance and the calculated capacity according to Safaei et al. [56] for the load resistance of 0.5 MΩ (maximum power output for experimental testing, see below). It was compared to the data for the experimental testing of the piezoelectric element only. These results were gathered by directly loading the piezoelectric element with the force profile, which was also used for the numerical calculation (also shown in Figure 8).

The voltage rose with the increasing force. When the load slope flattened and the force approached the first local maximum, the voltage dropped. It rose again with the second force peak. The unloading to the force minimum drove the voltage to negative values. This pattern was repeated for all cycles, with the first voltage maximum more pronounced in the initial cycle.

The two calculated voltage curves were in very good accordance regarding their course. However, the approximated capacity led to smaller absolute voltage values than for the nominal capacity from the manufacturer (e.g., the first maximum decreased by 22%). The difference between the experimental data and the data calculated on the basis of the nominal capacity from the manufacturer was even more pronounced (e.g., the first maximum decreased by 37%), but the trend matched very well.

**Figure 9:**
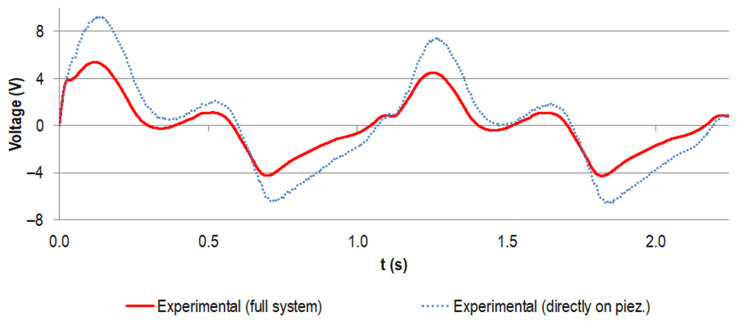
Experimental voltage data (V) for the first two gait cycles (time t in s) for the stacked configuration at a load resistance R of 0.5 MΩ. The curve for the implanted system is shown in red and the data for the directly loaded piezoelectric element are shown in blue (dotted line, same data as for Figure 8).

For the implanted system, the measured voltage at a load resistance of 0.5 MΩ is shown in Figure 9, together with the data for the directly loaded piezoelectric element (already known from Figure 8). The curve progressions were in very good accordance and the absolute values of the generated voltage for the implanted system were notably smaller. For example, for the first peak, the maximum was reduced by 46%.

**Figure 10:**
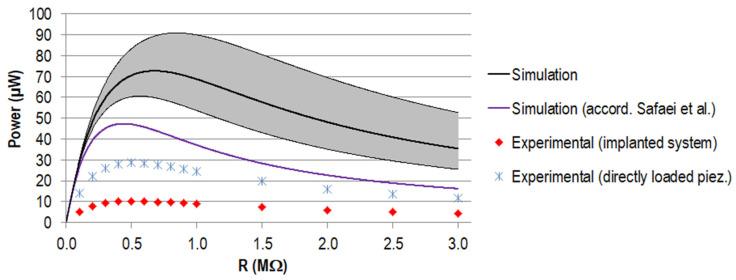
Generated power P (µW) for different load resistances R for the stacked configuration. Simulation data for the capacity provided by the manufacturer (in black, with a tolerance of ±20% in grey) and the capacity calculated according to Safaei et al. [56,57] (in purple). Experimental data are displayed for the implanted system (in red) and the directly loaded piezoelectric element in blue.

The generated power for different load resistances, calculated from the different voltage curves, is shown in Figure 10. The calculated power maximum amounted to 72.7 µW at 0.68 MΩ, assuming the nominal capacity from the manufacturer. When approximating the capacity according to Safaei et al. [56], the maximum was decreased by 35% to 47.4 µW and the corresponding resistance shifted to 0.44 MΩ.

The maximum power output for the experiment amounted to 28.6 µW for the directly loaded piezoelectric element and 10.2 µW for the implanted system, both for a load resistance of 0.5 MΩ. Regarding the data calculated on the basis of the nominal capacity from the manufacturer, this equalled a reduction of 61% and 86%, respectively. Between the directly loaded piezoelectric element and the implanted system, the difference was 18.4 µW (equals 65%). The curve progressions were all in accordance, showing a rise to the maximum power, followed by a slow decrease towards higher resistance.

## 4. Discussion

First, the numerical results of the simplified testing setup are discussed, along with the results from our sensitivity analysis. The second part focuses on our experimental data and relates them to the numerical simulations.

### 4.1. Finite Element Analysis: Deformation, Loading, and Sensitivity

#### 4.1.1. Reproduction of Results for New Test Setup

The first aim of this study was to reproduce the physiologically based loading situation from our previous complex FE model in a setup suitable for reproducible experimental testing, using an artificial bone segment in a simplified bearing and neglecting muscle forces. The qualitative deformation behaviour, i.e., displacement of point *C* and bending, was consistent with our previous simulation and with the physiological bending of the femur [41,59]. Furthermore, the stress distributions in the metallic implant component and in the piezoelectric element were similar. Regarding the local stress effects by the contact boundary conditions, the stress evaluation of the piezoelectric element’s undisturbed midplane served as a good representative value.

Our iterative study enabled us to quantitatively reproduce nearly all the output parameters. The remaining differences were acceptable, even for the notable relative differences that occurred for the stress within the piezoelectric element. Here, the absolute values of the differences were small (especially for the stacked configuration). To our knowledge, no fatigue data of PZT multilayer elements of comparable geometry, configuration, and loading for the purpose of energy harvesting have been published. However, the maximum values were still in the range of or below the literature data for stacked multilayer actuators tested with 20 MPa preload [60] or of typical pre-stressing of piezoelectric elements defined by the manufacturer [58]. The scaling of the force components and the resulting alignment of the artificial bone segment did not change the overall deformation behaviour [41,59]. The different scaling factors resulted mainly from the chosen loading and bearing, which differed from the physiologically based boundary conditions by using only a single force and reducing the model to a femur segment. Despite the simplified loading situation, the total force to reproduce a previous loading situation only needed to be scaled by 1.8% from in-vivo data.

The power approximation was improved with regard to our previous work. For the force input, more recent loading data from Bergmann et al. were used, since we did not include muscle forces that were only calculated by Heller et al. for the old data set [61]. Furthermore, the capacity of the piezoelectric element was not only calculated from its geometric properties and the material data (as done before following the approach of Safaei et al. [56,57]), but also with data from the manufacturer. This resulted in a much smaller capacity and higher power output (see Table 4 and Figure 10). As for our previous work, the power output for the stacked configuration was remarkably higher than for a single element. Further details regarding the overall power output are discussed together with the experimental data in Section 4.2. With our approach, we approximated the full gait cycle by scaling the load profile based on a single load step (instance of maximum hip contact force). In future work, especially when using the piezoelectric element as a self-powered load sensor, the extraction of the force profile F_33_ by a transient simulation may be considered. However, the simplification enabled us to significantly reduce the computational cost and perform the numerous simulations required in the sensitivity analysis and for the iterative study.

#### 4.1.2. Sensitivity Analysis

The results from the sensitivity analysis show that malalignment of the artificial bone segment can lead especially to highly varying output parameters. This implies that deviations in the experimental setup from the numerical model would contribute directly to differences between the measured data and the simulation data. On the contrary, the regarded results were highly unaffected by the embedding level. Regarding the implantation, the resection level (and hence the correct positioning of the implant and the energy harvesting system towards the resection level) and the thickness of the cement mantle were relevant for the generated power by directly influencing the transmitted force F_33_ on the piezoelectric element. Based on the results for the positioning of the whole artificial bone segment, we also hypothesize an influence on the power output from the alignment of the implant towards the bone segment, which will be analysed in further studies. The high sensitivity of the output parameters to the geometric inputs reveals the necessity of a very exact realisation of the resection, implantation, and alignment. Nevertheless, the complex geometry of the artificial bone will not allow for the complete reproduction of the virtual CAD model when cutting the femoral head, preparing the bone cavity, or placing the artificial bone segment in the specimen holder. To overcome this limitation, a reverse process would be possible. Starting with the implantation and embedding, the geometry can be reproduced virtually for FEA simulations based on imaging technologies. However, the effort of this approach must be weighed against the expected improvement in outcome.

Apart from the displacement of point *C* in vertical direction $d_{C,v}$ , the small or absent influence of the material data for the artificial bone on the output parameters is of large relevance, since the material was defined to be linear elastic, which idealises the anisotropic behaviour of the composite. Hence, these simplifications seem acceptable for this simulation. In contrast, the Young’s modulus of the metallic implant component notably influenced the strain gauge strain $\varepsilon_{\mathrm{SG}}$ and, along with stiffness of the UHMW-PE, the force and the power output, too. Thus, the two Young’s moduli values from the literature can result in discrepancies between simulation and experiment. To dispel this uncertainty, experimental testing (e.g., tensile tests) of the used material should be performed. Regarding further design optimisation, the sensitivity towards the materials’ stiffness implies that different material treatments or choice of materials can be used to increase the power output by improving the force transmission on the piezoelectric element. In this study, we did not extend our sensitivity analysis to the positioning of the strain gauge. From previous analyses, we know that malalignment can result in deviations in the lower single-digit percentage range [42].

Besides the explanation of possible differences between simulation and experiment (see Section 4.2.), the sensitivity analysis also sheds light on the mechanical properties and safety of the system. Regarding the metallic implant component, the stress σ_imp_ was for all configurations far below the literature fatigue data of 470 MPa for the same material at equal loading (maximum stress at 10^7^ cycles, 10 Hz, R = 0.1, in Eagle’s medium at 37 °C) [62] and below the maximum stress value of 417 MPa from previous simulations accompanying our endurance testing [42]. The maximum occurring stress was only 10.7 MPa higher than the overall maximum for the unmodified reference geometry for the physiologically based boundary conditions [41]. Since the reproduction of the loading situation was satisfactorily fulfilled, this outcome was expected. The prominent influence of the alignment on the stress σ_imp_ results from a change in the loading situation equal to a change in force components. This is also reflected by the adverse change of the stress in the cavity and the piezoelectric element compared to the acting contact force when rotating the artificial bone segment in the antero-posterior plane. For the same but scaled loading, correlation of all the output parameters could be expected; in contrast, different force components can lead to increased stress concentration, while the force transmission is impaired. Additional simulation or testing of the whole gait cycle with a multiaxial approach should be considered to identify further potential worst-case loading scenarios that were neglected by the simplified uniaxial setup used here.

The stress in the piezoelectric element σ_piez_ is affected by all the changed parameters of the energy harvesting system (Young’s moduli of the metallic component and UHMW-PE, position of the energy harvesting system towards the resection level, and the alignment). Even if the maximum 12% loading increase was only of an absolute value of 2.4 MPa and still in the tolerable ranges mentioned above, it is more critical than for the metallic implant component. Considering the influence of superposed parameters and considering other scenarios that may increase the transmitted force (e.g., more dynamic activities like running or stair walking, or higher body weight) more fatigue data for comparable piezoelectric elements and comparable loading situations is desirable for the evaluation. It must be ensured via further studies that the piezoelectric element can withstand all worst-case scenarios, either by assessment of the maximum acting forces or by advanced design solutions preventing overloads, e.g., by metallic counterparts blocking excessive cavity closure.

Further numerical investigations should focus on (patient) individual performance of the proposed energy harvesting system to study the applicability of the design. The small influence of the Young’s modulus of the artificial bone indicates a certain independence on the surrounding material; in contrast, the resection level and cement mantle thickness are presumable parameters that will vary intraoperatively. Additionally, the superposition of different configurations of our sensitivity analysis should be analysed. Considering different implantation variants (e.g., different implant stem sizes and head sizes, further geometries for resection, bone cavity and bone cement layer), different femora (e.g., size, bone density), different loadings (e.g., different body weight and multiaxial force input), and manufacturing tolerances is necessary for the further development of the proposed concept. This will be the basis for possible in-vivo testing.

### 4.2. Experimental Testing and Comparison to Numerical Data

#### 4.2.1. Model Validation

The validation of the FEA model by the quasi-static experiment showed excellent correlation regarding the coefficients of determination. The experimentally measured values of the strain gauge strain $\varepsilon_{\mathrm{SG}}$ and the displacement of point *C* in vertical direction $d_{C,v}$ were in the range of the magnitudes predicted by the FEA and are plausible concerning other studies [42,63]. In contrast to the strain, the deviation for the displacement $d_{C,v}$ between the numerical data and the experiment was not negligible. The force input can be eliminated as a reason, given the data from the load cell, which showed high accordance with the input data for the static and dynamic testing. The results from our sensitivity analysis may explain the deviations for the displacement, as mentioned in Section 4.1. The strong influence of the malalignment of the artificial bone segment towards the uniaxial testing machine can result in the determined differences, even if the uncertainties for the Young’s modulus of the artificial bone segment and the metallic implant component are ignored, which would lead to less stiff model behaviour. Since the influence on the displacement $d_{C,v}$ is distinctly more pronounced than on the strain $\varepsilon_{\mathrm{SG}}$ , the well-matching values for the latter are not in conflict. However, we cannot rule out compensating errors. For example, our previous studies showed smaller experimental strain values than for the FE model [42]. In the latter work, we also determined the malpositioning of the strain gauge as a relevant cause of deviations.

In the current study, the verification of the energy harvesting performance of the proposed concept was in focus. The significant effort to realise the experiment and the functional model allowed the testing of only a single sample. The X-ray of the implanted system showed qualitatively good accordance with the CAD model; however, further studies with an increased sample number should be considered, ideally for an optimised system [64]. Since we were not able to remove material from the cortical compartment with the rasp, the cement mantle varied in thickness and was partly incomplete, which is consistent with the CAD model. For further work with a more correct physiological representation, a homogeneous and fully enveloping cement mantle should be aimed for. The long-term objective of extended studies is to provide a system suitable for in-vivo testing.

#### 4.2.2. Interpretation of Experimental Data

The measured voltage curves for the implanted energy harvesting system were of lower magnitudes than the voltage data for the directly loaded piezoelectric element, yet the trends matched well (see Figure 9). This can be explained by a lower overall force level acting on the piezoelectric element in the implanted system. As our sensitivity analysis showed, the main reasons therefore could have been the malalignment of the artificial bone segment towards the uniaxial testing machine and differences between the realised and virtual implantation, e.g., a more proximal resection level and a thinner geometry of the cement mantle. The latter was visible in the radiographic inspection of the implantation. However, from these images, only qualitative comparisons were possible in the analysed plane. Again, the stiffness for the metallic implant component and the UHMW-PE housing are of relevance. Even though we did not specifically analyse a different alignment of the implant towards the femoral geometry, the results for the rotation in the antero-posterior and medio-lateral plane indicate a possible influence, since both effects change the proportion of the single force components. The geometric manufacturing tolerances of the piezoelectric element and the cavity and UHMW-PE housing can also influence the form fit and the force transmission. Even though pre-testing of the fully assembled system showed good results and no need for levelling or other measures to account for manufacturing tolerances between the piezoelectric element end faces and the housing [65], numerical studies accompanied by experiments can reveal the sensitivity towards geometric variations.

#### 4.2.3. Interpretation of Numerical Model and Relation with Experiments

Regarding the numerical voltage approximation, the measured data for the directly loaded piezoelectric element with similar force input were lower, revealing limitations of the numerical model used. The deviation was less pronounced when compared to the data calculated with the capacity on the basis of the geometry of the piezoelectric element, as done by Safaei et al. [56,57] and applied in our previous work [41]. However, this value must be questioned, since it differed from the capacity data provided by the manufacturer. The calculated capacity could be improved by considering passive top and bottom layers and passive margins, better representing the typical structure of piezoelectric multilayer elements. Due to the difference between the calculated and provided value, we recommend using the values specified in the data sheet or determining the capacity experimentally. Additionally, small differences between the experimental and numerical data can be linked to an imprecise d_33_ value of the PZT. However, we assume that the most significant influence resulted from model simplifications. For example, no energy dissipation term was part of the numerical model in Equation (3). In future work, the model should be studied to enhance its predictive power and establish better quantitative accordance with the experimental data. Depending on the specific problem, the model could be extended to better represent the piezoelectric behaviour. Model calibration can also be considered, promising a solution with less effort. This is shown in Appendix A.2. Based on a simple loading regime and experimental measurements, the numerical data could be fitted and the calibrated model could predict the voltage curves and power output for a more complex force profile.

Despite the differences between the quantitative numerical and experimental data in this work, the qualitative accordance and the trends within the frame of the sensitivity analysis were not affected. The voltage curve progression equals the findings of our previous work [41] and can be explained by the progression of the force curve and the time derivative of the force (see Equation (3)). The higher first initial voltage peak was due to the larger force difference when starting from pre-load only. In contrast to the force curve, in each load cycle, the second local peak did not reach the level of the first peak since the load was not completely removed and meanwhile, the voltage potential was decreased by draining current through the resistance. The latter effect also explains the negative voltage values in the last part of the cycle.

#### 4.2.4. Discussion of Power Output

All power curve progressions corresponded to the typical shape of graphs of generated power against load resistance, also found in other works on piezoelectric energy harvesting in load-bearing implants [27,28,41,56,64]. The location of the power maximum depended on the capacity of the piezoelectric element and was shifted towards smaller resistance values for a higher capacity, clearly visible for the different simulations and also for the data of a single element [41] or a customised geometry with comparable piezoelectric element structure [64]. The maximum calculated power for the stacked configuration differed from our previous work (31.1 µW) [41] despite a comparable maximum force level of F_33_ acting on the piezoelectric element’s end faces (see Table 5). This was mainly because we used a much lower input capacity provided by the manufacturer. A small difference was also present for the maximum power calculated according to Safaei et al. [56,57] since, for our previous work, we considered the passive top and bottom layers. Additionally, the actualised force profile from Bergmann et al. with more recent data had a slight influence [45].

Regarding the comparison of the quantitative data for the simulations and experiments, the different voltage levels described above were propagated directly to different power maxima. Since the voltage is contained as the squared value in the power calculation, the deviation was even more pronounced (cf. Equation (5)). Therefore, the differences between experiment and simulation and between the experimental setup of the directly loaded piezoelectric element versus the implanted system can be explained similarly, as discussed for the voltage on the basis of the sensitivity analysis (see above in Section 4.1.).

The maximum power generated experimentally for the implanted system (stacked piezoelectric element) was in the range of our calculation for the single piezoelectric element of our previous work [41]. During the experiment, the load resistance was changed by 0.1 MΩ increments in the region of the best matching resistance; for smaller increments, a slightly higher output might be found. The 2.8 times larger amount of generated power for the directly loaded piezoelectric element was notably higher and should be the envisaged output with the system at hand in further studies. Therefore, the reasons for the decreased force transmission between the simplified experiment and the implanted model must be identified; according to the sensitivity analysis, these are most probably malalignment or material properties.

The power output of our experimentally measured data was in the scale of the power output of other initially proposed concept variants for energy harvesting in load-bearing implants. Concerning THR, approximately 55 µW was reported for a multi-source energy harvesting system (two electromagnetic generators and a piezoelectric transducer) [66]. In a TKR application, Safaei et al. generated a raw power output of 5.5 µW to 12 µW for single and multiple piezoelectric multilayer elements [33,57,67]. These systems were optimised to generate more than 1 mW of power with an improved electromagnetic generator in THR [39,68] and 269.1 µW for four piezoelectric elements in TKR [34]. The power output of the system proposed by Platt et al., optimised by Almouahed et al., generated power of even more than 4 mW [27,30]. The latter energy harvesting systems will deliver enough energy for instrumented implants, even if the required amount strongly depends on the application. For low power applications in orthopaedic implants, e.g., sensory functions and data transmission, it was shown that power of <100 µW was sufficient [37,69]. Regarding implants with active functions, higher amounts of power are probably required, however, not necessarily continuously. For intermittent regimes, such as those proposed for the stimulation of bone growth [18,70], energy can be accumulated during the day and provided for the required periods. This is illustrated by the recently proposed implantable electrical stimulation unit for bone regeneration in the mandible [71]. The estimated power consumption for the low power mode was 0.17 mW and was increased to 0.67 to 2.56 mW during stimulation [71]. The mentioned power levels were not reached with our design at hand and were provided for the classification of our results in a broader context. In this study, we aimed for experimental verification of the energy harvesting performance of our recently proposed design [41]. Compared to the numerical investigations, the power output was of a comparable magnitude, but smaller than initially calculated [41]. Anyhow, higher power output is required for future applications. With the present proof of concept and using off-the-shelf piezoelectric elements, the basis for further experimental studies was provided. We have already numerically investigated the potential of increased power output for customised piezoelectric element geometries [64]: The cavity geometry of our proposed design, the usage of available space for piezoelectric element integration, and the force transmission were optimised to allow maximal power generation while maintaining the mechanical safety of the components. We found an increase of approximately factor 25 towards the comparable stacked piezoelectric configuration [64]. Applying this value to our experimental findings, we could generate 255 to 715 µW assuming the data from the implanted system or the directly loaded piezoelectric element. These amounts relate to raw generated power for optimal resistive loads. Additionally, multi-source energy harvesting with different systems is an approach for increasing the overall output, as proposed by Santos et al. [66]. In further studies, we will also consider energy harvesting circuitry including a voltage rectifier, voltage regulator, and a storage solution to determine a realistic efficiency. The harvested energy will be used for an arbitrary application, e.g., using the piezoelectric element as a self-powered load sensor by linking the generated voltage to the applied force.

## 5. Conclusions

This work aimed to experimentally verify the performance of a new concept for energy harvesting in THR. We therefore proposed a reproducible setup for the experimental testing of a modified total hip stem implanted in an artificial bone segment. In an iterative FEA study, the loading situation from our previous physiologically based model was reproduced [41]. The results from the sensitivity analysis shed light on the important factors for the system’s mechanical behaviour, mechanical safety, and the generated power output. This is also of relevance for further optimising the system and regarding patient individuality. The experimentally determined voltage and power data were in good accordance with the numerically calculated curve progressions. However, the absolute magnitudes of experimentally generated voltage and the maximum power were notably lower. A simplified testing setup of the piezoelectric element only revealed discrepancies towards the numerical model. Depending on the research question, the numerical model requires an extension to enhance its predictive power. A first step could be calibration, as shown in Appendix A.2.

In summary, the general feasibility of energy harvesting with the proposed concept was successfully demonstrated. The generated power output can be increased in future work, focusing on better use of the available space for the integration of piezoelectric elements. To enhance the applicability, the system will be investigated with energy harvesting circuitry, in combination with sensory or active functions that are powered by the energy harvesting system. The setup proposed herein can be used for experimental testing of the optimised design. Further, loading by a multi-axial joint simulator should be considered as the first step towards increased physiological boundary conditions. In future, the developed energy harvesting system must be verified in an in-vivo model.

## Figures and Tables

**Table 1:** Final force values in the femur-based coordinate system transferred from the iterative FEA study to experimental testing.

F_X_ (N)	F_Y_ (N)	F_Z_ (N)	F_total_ (N)
396.97	–167.82	–1843.27	1892.99

**Table 2:** Material properties.

Component (Material)	Young’s Modulus (GPa)	Poisson’s Ratio
Implant		
Metallic total hip stem (high-nitrogen stainless steel)	195 [49]	0.3 ^1^
Housing (UHMW-PE)	0.83 [50]	0.46 [50]
Piezoelectric element (PZT)	52.4 [51]	0.35 [51]
Bone cement (polymethylmethacrylate)	2.3 [52]	0.3 ^1^
Artificial bone		
Cortical bone (short fibre-filled epoxy)	16 [53]	0.26 [53]
Cancellous bone (polyurethane foam)	0.155 [53]	0.3 ^1^
Embedding resin (casting resin with filler)	2.4 [54]	0.3 ^1^

^1^ assumed values for Poisson’s ratio if no data available.

**Table 3:** Piezoelectric properties (relative permittivity and piezoelectric constants e and d) [51] ( $\varepsilon_{0}$ = 8.854 × 10^–12^ As/(Vm.)).

Parameter (Unit)	Value
$\varepsilon_{11}^{S}{/\varepsilon }_{0}\;$ (-)	936
$\varepsilon_{33}^{S}{/\varepsilon }_{0}$ (-)	759
$\varepsilon_{33}^{T}{/\varepsilon }_{0}$ (-)	1751
d_33_ (m/V)	3.996 × 10^–10^
e_31_ (N/Vm)	–6.730
e_33_ (N/Vm)	15.680
e_15_ (N/Vm)	13.140

**Table 4:** Piezoelectric element capacity in nF according to the manufacturer for a single element with ±20% tolerance [58] and calculated with Equation (2).

Configuration	Capacity acc. Manufacturer (nF)			Calculated Capacity (nF)
	Ref. Value	Low	High	
Single element	110	88	132	168.9
Stacked element	220	176	264	337.7

**Table 5:** Results of the iterative study to reproduce the implant and piezoelectric element loading situation from previous work [41] for single and stacked configurations. Final output values of the new loading situation with absolute and relative differences from the previous model.

			d_c,total_ (mm)	σ_imp_ (MPa)	σ_piez_ (MPa)	F_33_ (N)	V_OC_ (V)
**Single Element**	After iterative study		1.64	273.5	16.6	138.7	11.6
	Previous model [41]		1.65	269.4	14.1	141.6	11.9
	Difference	relative	–0.6%	1.5%	17.7%	–2.0%	–2.5%
		absolute	–0.01	4.1	2.5	–2.9	–0.3
**Stacked Element**	After iterative study		1.65	269.6	18.1	194.9	16.8
	Previous model [41]		1.65	267.2	16.9	196.3	16.9
	Difference	relative	0.0%	0.9%	7.1%	–0.7%	–0.6%
		absolute	0.0	2.4	1.2	–1.4	–0.1

**Table 6:** Initial maximum hip reaction forces based on Bergmann et al. [45] for walking by an average patient and final values after iterative study.

		F_X_ (N)	F_Y_ (N)	F_Z_ (N)	F_total_ (N)
Bergmann et al. [45]		535.72	–342.48	–1747.18	1859.28
Scaled after iterative study		396.97	–167.82	–1843.27	1892.99
Difference	relative	–25.9%	–51.0%	5.5%	1.80%
	absolute	–138.75	174.67	–96.09	33.71

**Figure A2:**
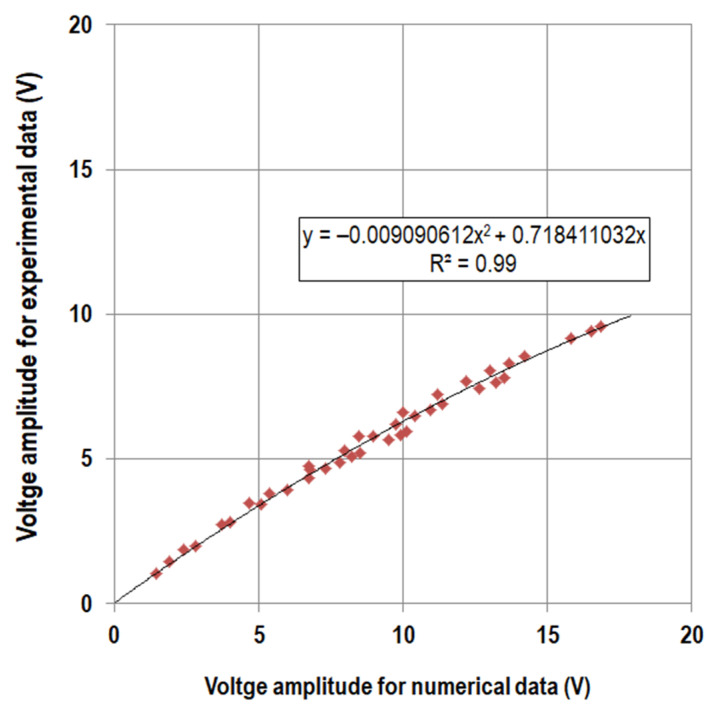
Voltage amplitudes (V) from sinusoidal fitting for numerically calculated data against amplitudes for experimental data and regression curve.

**Figure A3:**
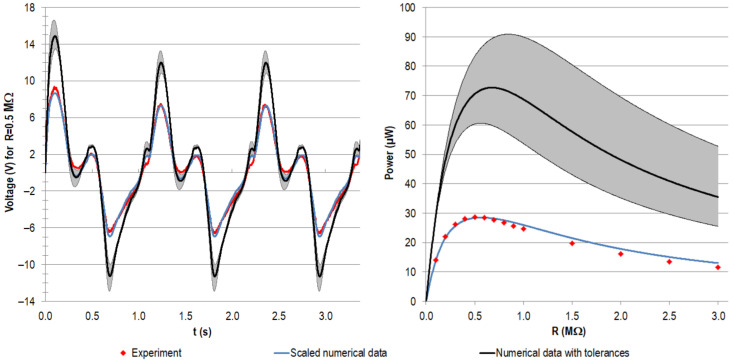
Exemplary voltage curves (V) for the first three cycles at R = 0.5 MΩ (**left**) and generated power output (µW) for variable load resistances (**right**). The initial numerical data (black) were scaled (blue) with the data from the regression model based on sinusoidal input. For the Bergman load profile, the scaled voltage matches the experimental data (red) notably better, resulting in high accordance with the power output.

## Data Availability

Data available on request.

## Appendix A

### Appendix A.2. Model Calibration

Based on a simple signal, we investigated the possibility of scaling and correcting the voltage in our numerical model. Therefore, we applied a sinusoidal force directly to the stacked piezoelectric element at three different force levels in the range of the expected actual maximum forces (15 to 150 N, 20 to 200 N, and 25 to 250 N, all at 1 Hz) and measured the generated voltage curves for 10 cycles for different load resistances (see Section 2. Materials and Methods). Accordingly, we calculated the generated voltage curves with our numerical approach (Equation (1)). For each numerical and experimental data set of 10 cycles, we fitted a sinusoidal function to the curves using MATLAB 8.4 R2014b and extracted the amplitude values. The numerical amplitude values were plotted against the experimental amplitude values and a quadratic regression analysis passing through the origin was performed (see Figure A2).

The regression model was used to scale the numerically calculated voltage curves for the Bergmann load profile with Equation (A1) below, assuming the contact force F_33_ from the FEA (194.93 N) and using the approach described in Section 2.1.6. Post-processing and Output Parameters. Values a and b were taken from the regression; the signum function was needed to account for the negative voltage values that would be lost by the square term.

(A1)
$$v_{scaled}\left(t\right)=\left(a*v{\left(t\right)}^{2}+b*\left|v\left(t\right)\right|\right)*sgn\left(v\left(t\right)\right)\;a=-0.009090612,\;b=0.718411032$$

From these data, we also calculated the power output. In Figure A3, the experimental and numerical voltage curves (original and scaled) are shown for the resistance of 0.5 MΩ. Additionally, the generated power output for variable load resistances is plotted.

The presented approach demonstrated the possibility of scaling the numerical model based on simple experimental input to improve the prediction of voltage and power output. The present study focused on the concept evaluation and the feasibility of energy harvesting. For numerical studies, where concrete output values are relevant, this procedure may be chosen.
