# Correction: Lange et al. Performance of a Piezoelectric Energy Harvesting System for an Energy-Autonomous Instrumented Total Hip Replacement: Experimental and Numerical Evaluation. *Materials* 2021, *14*, 5151

**DOI:** 10.3390/ma14247693

**Published:** 2021-12-13

**Authors:** Hans-E. Lange, Nils Arbeiter, Rainer Bader, Daniel Kluess

**Affiliations:** 1 Department of Orthopaedics, Rostock University Medical Center, 18057 Rostock, Germany; rainer.bader@med.uni-rostock.de (R.B.); daniel.kluess@med.uni-rostock.de (D.K.); 2 Institute of General Electrical Engineering, University of Rostock, 18059 Rostock, Germany; nils.arbeiter@uni-rostock.de

## Error in Figure

The authors wish to make the following corrections to their paper [1]. In the original publication, there was a mistake in Figure 4 as published. A conversion problem led to a corrupted left line in the circuit diagram. The corrected Figure 4 appears below.

## Text Correction

A correction has been made to Section 2.2.3. Mechanical Testing. There was an error in the original publication. In Equation (5), *V_Osc_* was used instead of *V_Piez_*:

(5) $$P=\frac{1}{T*n}\sum \frac{V_{Piez}{}_{}^{2}\left(t\right)}{R}t_{s}$$

Furthermore, there was an error introduced in the original publication after our proofreading. The text belonging to Appendix B was misleadingly placed in Appendix A. We reduced our Appendix to only one main section with two sub-sections and adopted the references in the main text.

A correction has been made to as follows:

The reference has been adopted in Section 3.1. Results of Finite Element Analysis: Deformation, Loading, and Sensitivity:

The results of the sensitivity analysis are described below, revealing the relevant input parameters influencing the output. The full result data are shown in Figure A1, Appendix A.1.

The results of the sensitivity analysis show that the contact force F_33_ and the open-circuit voltage V_OC_ were identically influenced; therefore, only the contact force F_33_ is presented and shown in Figure A1, Appendix A.1.

The reference has been adopted in Section 4.2.3. Interpretation of Numerical Model and Relation with Experiments:

Model calibration can also be considered, promising a solution with less effort. This is shown in Appendix A.2. Based on a simple loading regime and experimental measurements, the numerical data could be fitted and the calibrated model could predict the voltage curves and power output for a more complex force profile.

The reference has been adopted in Section 5. Conclusions:

Depending on the research question, the numerical model requires an extension to enhance its predictive power. A first step could be calibration, as shown in Appendix A.2.

A correction has been made to the Appendix A:

## Figures and Tables

**Figure 4:**
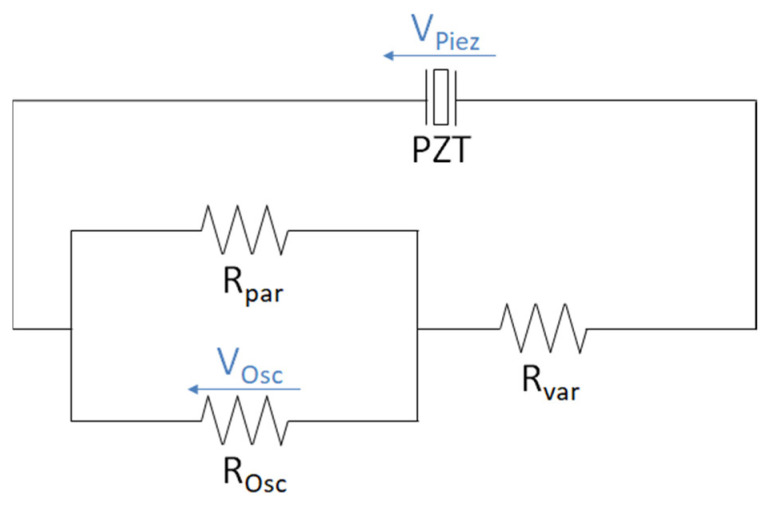
Measuring circuit of the piezoelectric element (PZT) for different load resistances with a voltage divider and an oscilloscope.

**Figure A1:**
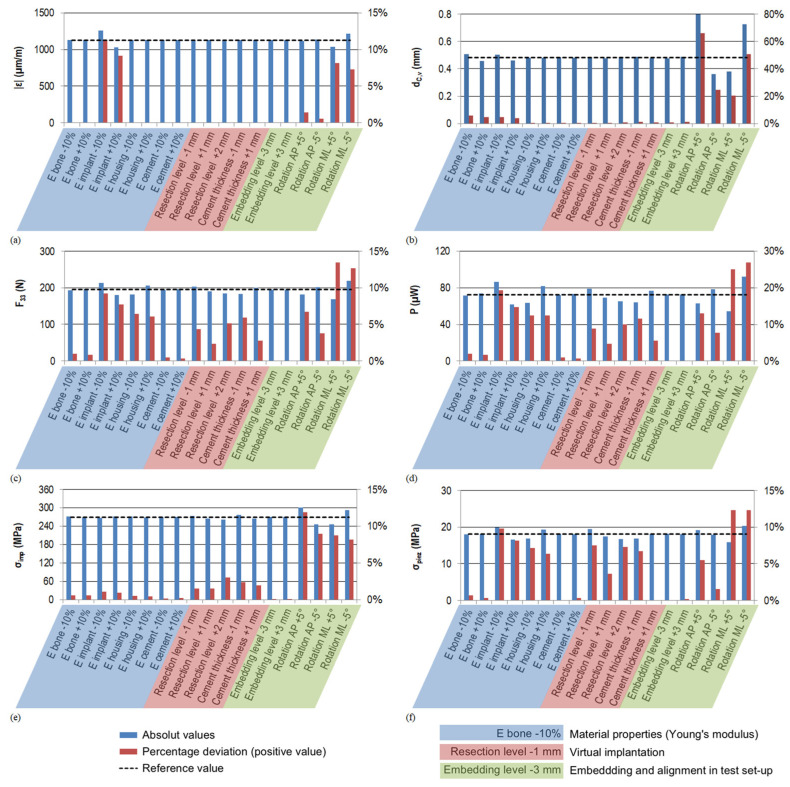
Results of the sensitivity analysis (absolute values in blue and percent deviation in red). The dashed line represents the original value of the reference model. For convenience, the absolute values of the percentages are shown. (**a**) Simulated strain for the strain gauge $\varepsilon_{\mathrm{SG}}\;$ (µm/m); (**b**) displacement of point C in vertical direction of the uniaxial testing machine (direction of the acting force) $d_{C,v}$ (mm); (**c**) contact force F_33_ acting on the piezoelectric element’s end faces in the direction of its cylinder axis (N); (**d**) generated power calculated from F_33_ (µW); (**e**) maximum von Mises stress in the implant cavity σ_imp_ (MPa); and (**f**) maximum von Mises stress in the piezoelectric element mid plane cross-section σ_piez_ (MPa). (Abbreviations: E—Young’s modulus; AP—antero-posterior; ML—medio-lateral).

**Figure A2:**
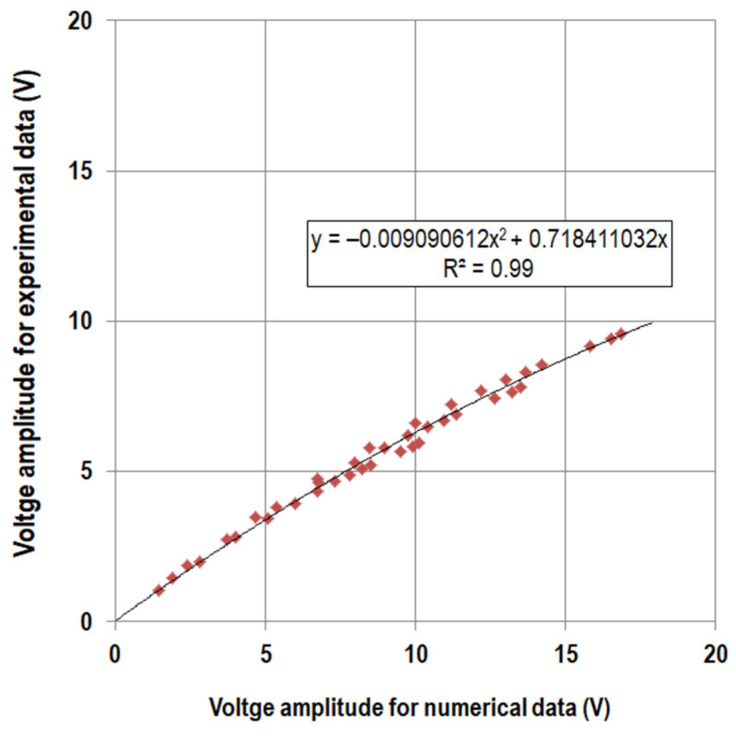
Voltage amplitudes (V) from sinusoidal fitting for numerically calculated data against amplitudes for experimental data and regression curve.

**Figure A3:**
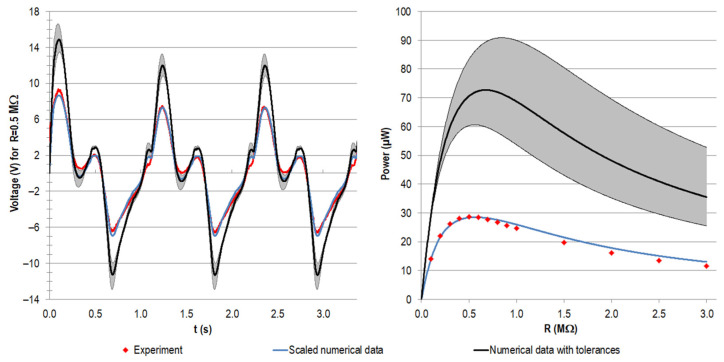
Exemplary voltage curves (V) for the first three cycles at R = 0.5 MΩ (**left**) and generated power output (µW) for variable load resistances (**right**). The initial numerical data (black) were scaled (blue) with the data from the regression model based on sinusoidal input. For the Bergman load profile, the scaled voltage matches the experimental data (red) notably better, resulting in high accordance with the power output.

## Appendix A

### Appendix A.2. Model Calibration

Based on a simple signal, we investigated the possibility of scaling and correcting the voltage in our numerical model. Therefore, we applied a sinusoidal force directly to the stacked piezoelectric element at three different force levels in the range of the expected actual maximum forces (15 to 150 N, 20 to 200 N, and 25 to 250 N, all at 1 Hz) and measured the generated voltage curves for 10 cycles for different load resistances (see Section 2. Materials and Methods). Accordingly, we calculated the generated voltage curves with our numerical approach (Equation (1)). For each numerical and experimental data set of 10 cycles, we fitted a sinusoidal function to the curves using MATLAB 8.4 R2014b and extracted the amplitude values. The numerical amplitude values were plotted against the experimental amplitude values and a quadratic regression analysis passing through the origin was performed (see Figure A2).

The regression model was used to scale the numerically calculated voltage curves for the Bergmann load profile with Equation (A1) below, assuming the contact force F_33_ from the FEA (194.93 N) and using the approach described in Section 2.1.6. Post-processing and Output Parameters. Values a and b were taken from the regression; the signum function was needed to account for the negative voltage values that would be lost by the square term.

(A1) $$v_{scaled}\left(t\right)=\left(a*v{\left(t\right)}^{2}+b*\left|v\left(t\right)\right|\right)*sgn\left(v\left(t\right)\right)\;a=-0.009090612,\;b=0.718411032$$

From these data, we also calculated the power output. In Figure A3, the experimental and numerical voltage curves (original and scaled) are shown for the resistance of 0.5 MΩ. Additionally, the generated power output for variable load resistances is plotted.

The presented approach demonstrated the possibility of scaling the numerical model based on simple experimental input to improve the prediction of voltage and power output. The present study focused on the concept evaluation and the feasibility of energy harvesting. For numerical studies, where concrete output values are relevant, this procedure may be chosen.

The authors apologize for any inconvenience caused and state that the scientific conclusions are unaffected. The original publication has also been updated.
